# Integrating Fluorescent Nanodiamonds into Polymeric Microstructures Fabricated by Two-Photon Polymerization

**DOI:** 10.3390/nano13182571

**Published:** 2023-09-16

**Authors:** Filipe A. Couto, Marcelo B. Andrade, Adriano J. G. Otuka, Sebastião Pratavieira, Sergio R. Muniz, Cleber R. Mendonça

**Affiliations:** São Carlos Institute of Physics, University of São Paulo, P.O. Box 369, São Carlos 13560-970, SP, Brazil; filipe.couto@usp.br (F.A.C.); mabadean@usp.br (M.B.A.); adriano.otuka@unesp.br (A.J.G.O.); prata@ifsc.usp.br (S.P.); srmuniz@ifsc.usp.br (S.R.M.)

**Keywords:** two-photon polymerization, nanodiamonds, quantum emitters

## Abstract

Nitrogen-vacancy (NV) and other color centers in diamond have attracted much attention as non-photobleaching quantum emitters and quantum sensors. Since microfabrication in bulk diamonds is technically difficult, embedding nanodiamonds with color centers into designed structures is a way to integrate these quantum emitters into photonic devices. In this study, we demonstrate a method to incorporate fluorescent nanodiamonds into engineered microstructures using two-photon polymerization (2PP). We studied the optimal concentration of nanodiamonds in the photoresist to achieve structures with at least one fluorescent NV center and good structural and optical quality. Fluorescence and Raman spectroscopy measurements were used to confirm the presence and location of the nanodiamonds, while absorbance measurements assessed scattering losses at higher concentrations. Our results show the feasibility of fabricating microstructures embedded within fluorescent nanodiamonds via 2PP for photonics and quantum technology applications.

## 1. Introduction

Some fluorescent defects in diamonds, known as color centers, have attracted significant attention in quantum optics and sensing due to their unique properties. Among them, NV-color center (hereafter referred to as the NV center) is a particularly promising platform for many applications due to its many favorable features, such as a spin state that can be optically set and read out [1], single-photon emission at room temperature, long coherence time, and photo-stability, since it does not present photon-blinking or photon-bleaching [2,3]. Furthermore, among various other uses, these color centers have been employed to mark cells in biological applications [4,5] and in many studies related to quantum information processing [6,7,8].

Since many applications in quantum optics and in quantum information processing, often require the integration of these emitters into photonic structures capable of confining, guiding, and enhancing their emission [9], much efforts have been made to incorporate these color centers in photonic devices such as photonic crystals [10], waveguides [11,12], and resonant cavities [13,14]. One alternative is to fabricate these photonic structures directly within bulk diamonds and then carefully place the NV center at desired positions. However, fabricating microstructures within bulk diamonds presents technical challenges and limitations due to the strong bond between carbon atoms, making diamonds one of the hardest materials available. A solution to this difficulty is embedding nanodiamonds (NDs) with NV centers into more easily microfabricated structures to integrate these color centers into designed photonic structures. An added advantage is that, in principle, the same technique could be applied to a variety of host materials, such as SiC [15], SiO_2_ [16], and GaP [17]. A remaining challenge is that, despite much progress, positioning the nanodiamonds inside the volume of the structures remains challenging [15]. A consequence of this limitation is that the interaction with the color centers occurs mainly with the evanescent field of the devices.

To expand the variety of materials and techniques to promote the integration of nanodiamonds into photonic structures, we propose a new approach using two-photon polymerization (2PP) as a promising technique to incorporate the color centers into designed photonics structures. Two-photon polymerization is a direct laser writing (DLW) method that exploits the nonlinear absorption of a photoresist to produce structures with sub-diffraction resolution and nearly arbitrary shapes. Due to the high confinement of the nonlinear interaction, the 2PP technique is well-suited to fabricate photonic structures capable of operating in the optical and near-infrared spectral ranges. Its sub-diffraction resolution permits the resolution of critical features that can be a source of optical losses, such as surface roughness, a challenge that would be difficult to address with methods that rely on linear absorption. It has been used to create a variety of optical structures for applications in many fields, such as photonic crystals [18], waveguides [19], resonators [20], and microneedles [21]. One of the main advantages of the 2PP technique is that it is relatively simple to incorporate dopants into the photoresist [22], making 2PP an appropriate method to fabricate functional structures, such as nanotube-doped structures [23], rhodamine-doped, and graphene oxide-doped microresonators [24,25].

This work uses a photoresist doped with fluorescent NDs to produce acrylate polymeric microcylinders embedded with NV color centers via the 2PP technique. The fabricated cylinders are 50 µm high and have a radius varying from 15 µm to 30 µm. Various concentrations of NDs were tested, and for proportions of 0.005 wt% and below, a favorable trade-off between the number of emitters and the structural quality of the resulting cylinders was observed. In addition, fluorescence spectroscopy was used to localize the fluorescing NDs in the structures, and Raman spectroscopy measurements were used to confirm the presence of the nanodiamonds in those specific locations within the structures. This work provides a comprehensive study of the fabrication process of structures embedded within NV color centers via 2PP, paving the way to leverage the versatility of this 3D direct laser writing technique with the incorporation of an important solid-state emitter that can be employed in a range of applications, from sensing to quantum information.

## 2. Materials and Methods

The photoresist consists of a mixture of dipentaerythritol pentaacrylate (SR399—Sartomer^®^, Exton, PA, USA) and tris(2-hydroxy ethyl)isocyanurate tryacrylate (SR368—Sartomer^®^) monomers, at a ratio of 90/10, and 3% additional weight of Lucirin TPO-L as a photoinitiator. A solution of nanodiamonds in deionized water at a concentration of 1 mg/mL was incorporated into the monomers and photoinitiator, and the whole mixture was submitted to magnetic stirring at 50 °C until homogenization and complete evaporation of the deionized water. The mean diameter of the nanodiamonds is 40 nm, as informed by the vendor (Adámas Nanotechnologies, Raleigh, NC, USA). The nanodiamonds’ photoluminescence (PL) properties are also reported by the supplier [26].

The two-photon polymerization setup consists of a Ti:Sapphire oscillator delivering 100 fs pulses centered at 780 nm with an 86 MHz repetition rate. The laser is sent through a half-wave plate and a polarizer to control the intensity, then to a pair of galvanometric mirrors, and finally to a 0.25 NA microscope objective that focuses the beam into the sample. To perform the fabrication, dedicated software controls the galvanometric mirrors, defining the x and y positions of the beam, and a motorized stage that sets the z position of the sample. More details about the fabrication process are given elsewhere [22,23,24,25].

The nanodiamonds were localized/observed using a commercial confocal laser scanning microscope (LSM) from Zeiss, Jena, Germany (model LSM-780) under laser excitation at 543 nm.

**Figure 1:**
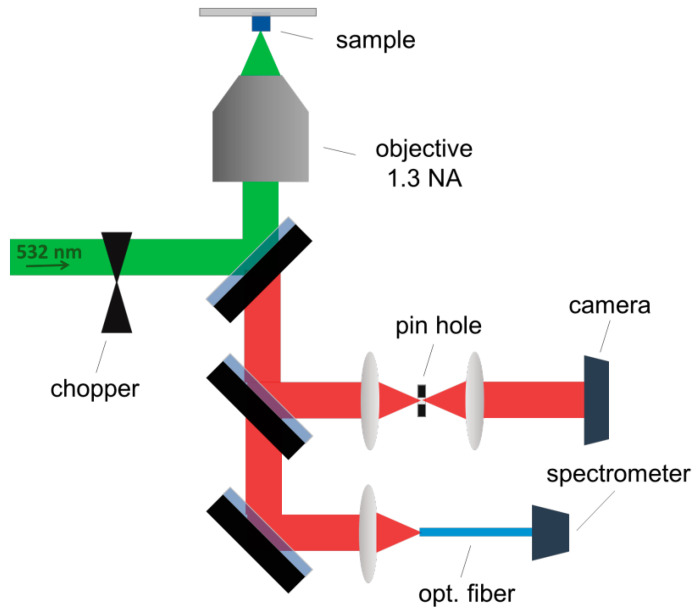
Schematics of the setup used to localize the nanodiamonds and measure the fluorescence of the NV color centers in the microstructures.

The emission spectra of the nanodiamonds were measured with a homemade fluorescence spectroscopy setup. A microscope objective was used to excite the sample with a diode laser emitting at 532 nm and to collect the nanodiamond fluorescence. A dichroic mirror separated the excitation light from the fluorescence, which was then sent through a piece of glass that works as a beam splitter. The Fresnel reflection was directed to a CMOS camera to obtain a real-time image of the sample. Before reaching the camera, a pinhole was placed at the focus of the signal to establish a confocal configuration. The signal transmitted through the glass was focused on an optical fiber, which was either connected to a spectrometer for fast analysis or to a monochromator with 1800 lines/mm for higher spectral resolution. The latter sent the spectrally separated light to a photomultiplier tube. A schematic of this setup is presented in Figure 1.

The presence of nanodiamonds in discrete positions of the structures was confirmed through Raman and photoluminescence (PL) measurements using a LabRAM equipment with 532 nm and 633 nm excitation wavelengths and a 100× objective to focus the laser onto the samples.

## 3. Results

**Figure 2:**
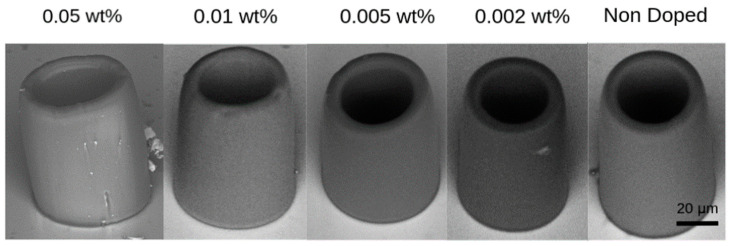
SEM micrography of cylindrical microstructures fabricated with photoresists doped with different proportions of nanodiamonds.

We conducted a study to assess the quality of fabricated structures based on the concentration of nanodiamonds in the photoresist. To do so, we used scanning electron microscopy (SEM) to qualitatively analyze the fabricated cylinders. Our results indicate that concentrations of 0.5 wt% did not result in successful fabrication via the 2PP technique. We believe that this upper concentration limit for successful fabrication arises from the nature of the two-photon absorption phenomenon, which scales with the square of the electric field intensity. Hence, the scattering losses induced by the nanoparticles at concentrations of 0.5 wt% and above are high enough to attenuate the laser intensity to values below the polymerization threshold within the volume of the photoresist. For concentrations ranging from 0.01 wt% to 0.05 wt%, fabrication was possible, but the final structures were poorly formed and had a high number of agglomerates, as can be seen in Figure 2. This can negatively impact their performance in photonics and quantum optics due to excessive light scattering. However, we found that proportions lower than 0.01 wt% produce clean and well-formed cylindrical structures with good structural and optical quality. As can be seen in Figure 2, for concentrations ranging from 0.01 wt% and below, the structures closely resemble the non-doped one.

**Figure 3:**
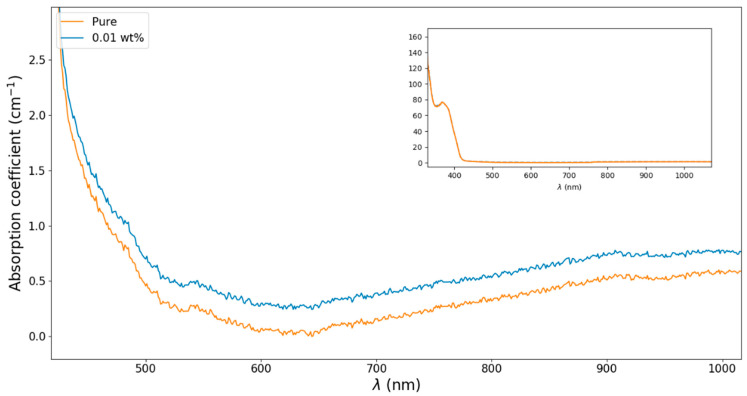
Absorption coefficient of the pure photoresist (orange line) and for the photoresist doped with nanodiamonds at the proportion of 0.01 wt% (blue line).

We also investigated the losses caused by the presence of nanodiamonds in the photoresist to assess the effect of light scattering. For this purpose, we used absorbance measurements on 400 µm thickness films of the photoresist cured in UV light, doped with a concentration of 0.01 wt%, which is the observed upper limit to fabricate resonators with good structural quality. To determine the absorption coefficient, we used the Beer–Lambert law. As shown in Figure 3, our results indicate that the doped photoresist had an overall offset of around 0.4 cm^−1^ compared to the pure photoresist. This offset was present even in the spectral region where no absorption was expected. Also, features of the absorption spectra of the nanodiamonds [26] cannot be identified, indicating that scattering induced by the nanoparticles is the main source of the extra losses. The total Q factor of a resonator is given by $\frac{1}{Q}={\left(\frac{1}{Q_{s.s.}}+\frac{1}{Q_{abs}}+\frac{1}{Q_{rad}}\right)}^{-1}$, where $Q_{s.s.}$ accounts for losses induced by surface scattering, $Q_{rad}$ accounts for radiative losses, and $Q_{abs}$ accounts for material absorption losses. The latter can also be given as a function of material absorption as $Q_{abs}=\frac{2\pi n}{\lambda_{0}\alpha }$, where α is the material coefficient absorption [25]. Since the quality factor of a typical non-doped microresonator, fabricated with the same photoresist, is on the order of $10^{5}$ [25], it is possible to estimate that the extra material losses induced by the nanoparticles would reduce the quality factor to $3\times 10^{4}$ in the spectral region of the NV center emission. Hence, the extra material absorption due to the dopant will not prevent these structures to be used in photonics applications.

**Figure 4:**
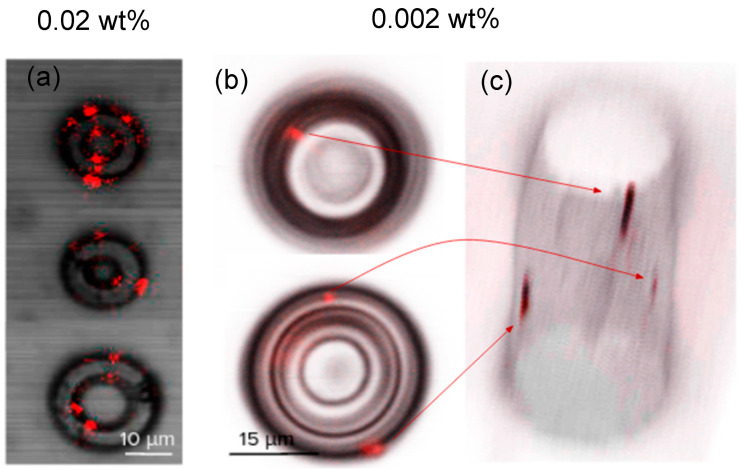
(**a**) Confocal scanning image of three structures doped with 0.02 wt%, where it is possible to observe several fluorescent spots for a single confocal plane. (**b**) Two different planes of a structure doped with 0.002 wt%; three fluorescent spots in different focal planes are observed. (**c**) A 3D image composition of the structure depicted in (**b**), where it is possible to observe the three fluorescent spots in the volume of the cylinder. The arrows illustrate the correspondence of the fluorescent spots between the plane image (**b**) and the 3D rendering (**c**). Fluorescent points were artificially colored based on the filter used.

The commercial LSM was used to sweep the structures and map the positions where the NV color center fluorescence was located to estimate the position and number of emitters in the structures. For concentrations of 0.01 wt% and above, fluorescent spots could be easily found in many locations of the structures. Figure 4a shows a scanning image, using a 10× microscope objective, of three different structures doped with a concentration of 0.02 wt% of nanodiamonds, where it is possible to locate many fluorescent points (artificially colored based on the filter used) are visible in a single scanning plane. For concentrations of 0.005 wt% and below, almost all studied structures presented one to three emitters embedded within their volume. Therefore, 3D scanning was often necessary to localize the fluorescent points. Figure 4b shows an example of a structure doped with a concentration of 0.002 wt%, in which three fluorescent spots were identified in different planes of the cylinder, as observed in the 3D scanning image (Figure 4c). Therefore, for many applications, concentrations of 0.005 wt% and 0.002 wt% may be the best options since they present a good chance of finding at least one emitter per structure, while maintaining good structural quality and low absorption coefficients across a broad spectral region.

**Figure 5:**
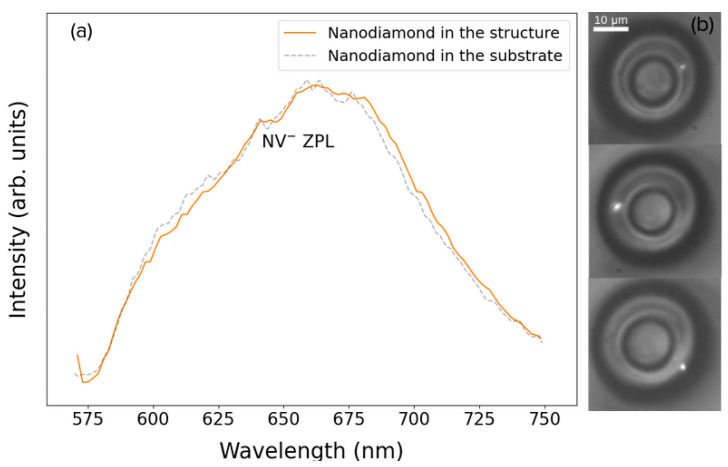
(**a**) Fluorescence spectra of an NV center inside a structure (solid line) and dispersed in a glass substrate (dashed line). (**b**) Optical images of the nanodiamonds when excited in the confocal fluorescence setup. A bandpass filter is used in the CCD camera in order to cut out the scattered light from the pump laser.

To further confirm the presence of nanodiamonds, the homemade confocal setup described in Figure 1 was used to sweep the structures’ volume and measure the bright points’ fluorescence spectra. The results for one structure doped with 0.002 wt% are depicted in Figure 5, where three fluorescent spots (bright white spots) were found in the volume of the structure (Figure 5b), and the NV center’s characteristic spectra are seen in Figure 5a.

**Figure 6:**
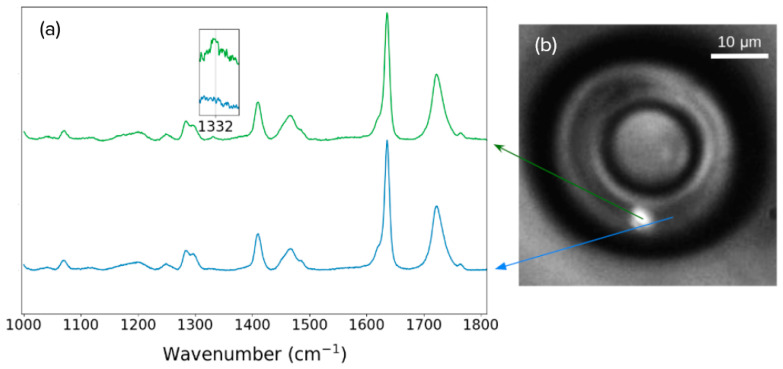
(**a**) Raman spectra for two points in a cylinder doped with 0.002 wt% of nanodiamonds. The inset displays a zoomed-in view of the diamond peak at 1332 cm^−1^, visible only at the point where the fluorescent is found. (**b**) Image from the setup depicted in Figure 1, while the fluorescent nanodiamond is excited with a 532 nm laser source focused with a 1.3 NA microscope objective, illustrating the positions in which the Raman spectra were measured.

In addition, Raman spectroscopy measurements were carried out on a previously characterized cylinder, doped with a concentration of 0.002 wt%. The Raman spectra are shown in Figure 6, with excitation set at 633 nm to avoid interference from the NV centers’ fluorescence. As depicted in Figure 6b, the measurements were performed at two different locations: one where a fluorescence spot was previously found (green line—Figure 6a) using the confocal setup (depicted in Figure 1), and another in a neighboring location where no fluorescence was detected (blue line—Figure 6a). Figure 6b illustrates the positions in which each Raman spectrum was measured. As seen in Figure 6a, the Raman spectra are similar, except for a small peak at 1332 cm^−1^ (magnified in the inset of Figure 6a), which is characteristic of the pristine diamond atomic lattice [27]. This peak is only visible at the point where the fluorescence was previously found.

**Figure 7:**
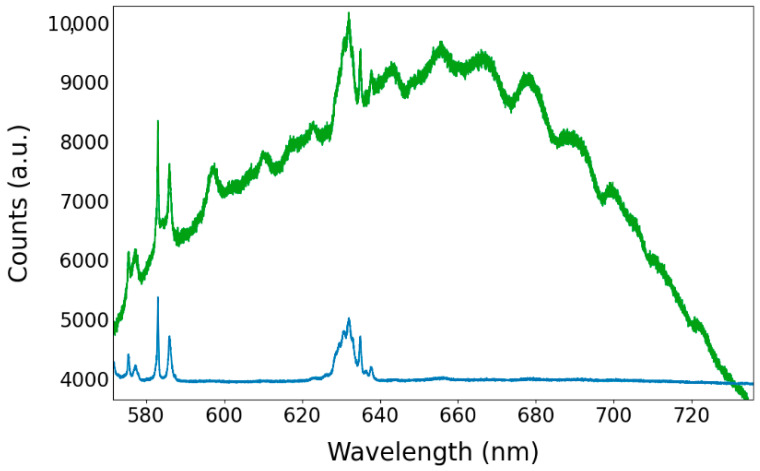
Photoluminescence measures carried out with the LabRAM equipment at two different points within the structure. Only in the position where the Raman signal of the diamond was found (green line) is the PL signal of the NV center observable as a baseline, alongside the Raman signal of the host material.

The results obtained using an excitation wavelength of 532 nm, which falls within the absorption spectrum of the NV color center, are depicted in Figure 7. In this figure, the NV PL spectrum serves as a baseline only in the points where the Raman signal of the diamond is observable (green line) and is absent in the neighboring point (blue line). Figure 7 illustrates the broad background that corresponds to the NV center emission, similar to the one presented in Figure 5, as well as the Raman peaks of the polymeric resin (575, 577, 583, 625–639 nm).

Hence, data obtained by the Raman and PL measurements further confirm the presence of fluorescent nanodiamonds in discrete points within the volume of the structures while adding low material losses.

## 4. Discussion

This investigation has shown that the fabrication of clean and well-formed cylindrical microstructures doped with nanodiamonds via 2PP is achievable for proportions lower than 0.01 wt%. However, concentrations ranging from 0.01 wt% to 0.05 wt%, result in poorly formed structures with many agglomerates, compromising their suitability for photonics and quantum optics applications. We were able to produce microstructures containing one to three fluorescing nanodiamonds using concentrations ranging from 0.005 wt% to 0.002 wt%. This concentration range strikes a good balance between the probability of finding at least one emitter per structure while also maintaining excellent structural quality and low absorption coefficients across a broad spectral region. Additionally, Raman and PL measurements confirm the presence of these fluorescent nanodiamonds at specific positions within the structures.

Although this technique permits producing structures with incorporated NV centers within their volume, it is still not possible to precisely control where the nanodiamonds will be positioned. Nevertheless, this work contributes to integrating NV centers into photonic polymeric structures. It paves the way for future research to investigate the interaction of NV centers with polymeric structures, creating a platform suitable for quantum optics applications. The sharp resonances of the cylindrical cavities can be explored to collect single photon emissions from the NV centers in precise spectral regions that can be tuned by varying geometric parameters of the resonators. Furthermore, it is worth mentioning that the results obtained for cylindrical structures can be directly applied to produce other devices with different geometries, such as waveguides, which can be used to launch the NV center’s emission in photonic circuits and interferometers, with possible applications in quantum information processing. Additionally, other nanoparticles containing different color centers in nanodiamonds, such as SiV, can be readily explored using the same methods presented in this paper. Also, our findings can serve as a starting point for achieving integration with other types of emitters, which can be explored using similar procedure as those presented in this work.

## 5. Conclusions

In this work, we demonstrate the feasibility of micrometric structures doped with nanodiamonds containing NV color centers, presenting good structural quality and a controllable number of emitters per structure. This work paves the way for exploring the 2PP technique to produce photonics structures embedded with this important solid-state emitter, which can be used in applications in sensing and quantum optics.

## Data Availability

The data presented in this study are available on request from the corresponding author.
